# Effects of Elevated Oxytetracycline on Cytochrome P450 and Immune-Related Gene Expression and Histopathological Alterations in Olive Flounder, *Paralichthys olivaceus*

**DOI:** 10.3390/ani16121853

**Published:** 2026-06-16

**Authors:** Hyeon Ju Na, Gi Baeg Lee, Hee Jeong Kong, Ju-Won Kim, Seong Don Hwang

**Affiliations:** 1 Department of Convergence Study on the Ocean Science and Technology, Korea Maritime & Ocean University, Busan 49112, Republic of Korea; 2 Department of Convergence Interdisciplinary Education of Maritime & Ocean Contents, Korea Maritime & Ocean University, Busan 49112, Republic of Korea; 3 Genetics and Breeding Research Center, National Institute of Fisheries Science, 81-9, Geoje-si 53334, Republic of Korea; heejkong@korea.kr; 4 Division of Convergence on Marine Science, Korea Maritime & Ocean University, Busan 49112, Republic of Korea

**Keywords:** olive flounder, oxytetracycline, cytochrome P450, drug metabolism, immune response, histopathology

## Abstract

Oxytetracycline (OTC) is widely used in aquaculture to treat bacterial diseases, but its effects on drug metabolism and immune responses remain poorly understood in olive flounder (*Paralichthys olivaceus*). This study investigated the effects of OTC immersion for 1 h at different concentrations on the expression profile of drug metabolism- and immune-related genes and the histopathological changes in the gills and liver of olive flounder. The expression of *Paralichthys olivaceus* cytochrome P450 *(PoCYP*), *Paralichthys olivaceus* diphosphate (UDP)-glucuronosyltransferases (*PoUGTs*), and immune-related genes significantly increased at 12 and 24 h and then decreased at 72 h in the 100 and 200 ppm groups. By contrast, the 400 ppm group exhibited variable expression patterns depending on the gene and tissue. The highest expression levels were observed for *PoIL-1β* in the gills (22.7-fold) and *PoCYP27B1* in the liver (58.0-fold) in the 400 ppm group. Histopathological analysis revealed macrophage activation in the liver at 72 h in the 200 ppm group, whereas epithelial edema and mild aneurysmal changes in the gills and macrophage activation in the liver were observed in the 400 ppm group. These findings provide insights into the effects of OTC exposure on drug metabolism and immune responses in olive flounder.

## Simple Summary

Olive flounder is an important aquaculture species in South Korea, and antibiotics such as oxytetracycline (OTC) are widely used to control bacterial diseases. In this study, we investigated the effects of OTC immersion at different concentrations. Our results showed that OTC exposure increased the expression of drug metabolism- and immune-related genes in the gills and liver, particularly at early time points. In addition, histopathological alterations were observed at higher concentrations of OTC. These findings provide useful information for understanding the drug metabolism and toxicity effects of OTC in olive flounder and may help improve its safe use in aquaculture.

## 1. Introduction

Olive flounder (*Paralichthys olivaceus*), an economically important aquaculture species, is the most highly produced finfish species in Korea. Since the 1980s, the olive flounder aquaculture industry has expanded rapidly with the development of aquaculture and artificial seed production technologies [1]. However, the various diseases reported in olive flounder aquaculture every year have caused substantial economic losses. Bacterial disease outbreaks [2] have led to the widespread use of antibiotics for disease control.

Oxytetracycline (OTC) is a broad-spectrum tetracycline antibiotic widely used in Korean aquaculture to control bacterial diseases by inhibiting bacterial protein synthesis at the ribosome [3,4]. The appropriate use of antibiotics is important to minimize potential adverse effects on cultured fish and the aquaculture environment. Once absorbed into fish tissues, antibiotics undergo metabolic processes that may alter physiological and immune responses. Antibiotic exposure has been shown to influence the expression of genes involved in drug metabolism in fish [5]. In fish, xenobiotics such as antibiotics are primarily absorbed through the gills and subsequently transported to different tissues via the bloodstream [6]. Once absorbed, these compounds are delivered to metabolic organs, particularly the liver, which plays a central role in the detoxification of foreign compounds [7,8]. These detoxification processes occur as part of drug metabolism, in which xenobiotics are enzymatically modified to facilitate excretion and reduce pharmacological activity [9].

Drug metabolism is generally divided into Phase I and II reactions. Phase I reactions involve enzymes such as cytochrome P450 (*CYP*), which directly modifies drugs or generates metabolic intermediates. Approximately 94 *CYP* genes have been identified in zebrafish (*Danio rerio*), a widely used experimental model species for drug metabolism studies [10]. These genes include the *CYP1* family involved in detoxification and elimination processes, the *CYP2* family induced by xenobiotic and endogenous compounds, and the *CYP27* family associated with cholesterol metabolism [11,12]. In Phase II reactions, modified drugs are conjugated with polar molecules by enzymes such as uridine diphosphate (UDP)-glucuronosyltransferases (*UGTs*), thereby facilitating drug inactivation and excretion [5,13,14].

Previous studies have reported that excessive antibiotic exposure can induce histopathological alterations in fish organs. High-dose florfenicol administration caused hepatomegaly, renal tubular degeneration, hydropic swelling, and necrotic lesions in Nile tilapia [15]. Antibiotics used in aquaculture may persist in fish tissues for a certain period even after a single exposure and may affect immune responses in olive flounder [16]. Although several studies have reported the pharmacokinetics and physiological impacts of OTC exposure in olive flounder [17,18,19], studies focusing on the molecular responses to OTC exposure, particularly drug metabolism-related and immune-related responses, remain limited. Therefore, comprehensive evaluation of antibiotic exposure should include both drug metabolism and immune responses. Among immune-related factors, cytokines play important roles in regulating inflammatory and immune responses in fish [20].

Olive flounder is the most widely cultured fish species in Korea. However, standardized immersion concentrations of OTC for this species have not yet been clearly established in Korea. Therefore, this study aimed to investigate the effects of exposure to different concentrations of OTC on the expression of drug metabolism-related and immune-related genes and histopathological patterns in the gills and liver of olive flounder.

## 2. Materials and Methods

### 2.1. Experimental Animals and Immersion

Olive flounder with an average total length of 14.3 ± 1.5 cm and an average body weight of 24.8 ± 2.0 g were purchased from a fish farm in Hadong-gun, Gyeongsangnam-do, Korea. The fish were acclimated for 2 weeks at a density of nine fish per tank (68 L) at 20 ± 1.5 °C and fed a commercial diet once daily. The experiment was conducted for 3 days, and the water temperature was maintained at 20 ± 1.5 °C throughout the experimental period. Water was exchanged daily in all experimental tanks throughout the experimental period. The 100 ppm concentration of OTC was selected as the reference dose based on the recommended immersion range (approximately 50–100 ppm) in red sea bream [21], and 200 and 400 ppm were set as 2- and 4-fold higher doses, respectively, to evaluate the effects of elevated OTC exposure in olive flounder. The fish were then exposed for 1 h to nominal concentrations (100, 200, and 400 ppm) of the commercial OTC formulation (Hitmycin-24, Handong Co., Seoul, Republic of Korea) diluted in the exposure water, after which the exposure water was replaced with fresh seawater. The control group was not exposed to OTC. After the immersion, three fish were randomly sampled from each control and experimental group at 12, 24, and 72 h. Individual fish were considered biological replicates for gene expression and histopathological analyses. The experimental fish were anesthetized with MS-222 (ethyl 3-aminobenzoate methanesulfonate salt, Sigma-Aldrich, St. Louis, MO, USA) at a concentration of 100 ppm and gills and liver tissues were collected. All fish experiments were approved by the Institutional Animal Care and Use Committee of Korea Maritime and Ocean University (KMOU IACUC 2024-02).

### 2.2. Gene Expression Analysis Using Real-Time PCR

Total RNA was extracted from the gills and liver using an RNeasy Plus Mini Kit (Qiagen, Hilden, Germany) in accordance with the manufacturer’s instructions. cDNA was synthesized from total RNA using a TOPscript™ cDNA Synthesis Kit (Enzynomics, Daejeon, Republic of Korea) and stored at –80 °C until use. Real-time PCR was performed in a total volume of 20 μL containing 1 μL of cDNA, 10 μL of TOPreal SYBR Green qPCR PreMIX (Enzynomics, Daejeon, Republic of Korea), 7 μL of distilled water, and 1 μL each of forward and reverse primers (Table 1). Drug metabolism-related genes (*PoCYP1A1*, *PoCYP1B1*, *PoCYP2B4*, *PoCYP27B1*, and *PoUGT*) and immune-related genes (*PoIL-1β*, *PoIL-6*, *PoIL-8*, and *PoTNF-α*) were selected for expression analysis. The amplification procedure included an initial denaturation step at 95 °C for 10 min, followed by 40 cycles of 95 °C for 10 s, 60 °C for 1 min, and a final melting curve analysis at 95 °C for 1 min, 60 °C for 15 s, and 98 °C for 5 s. β-actin was used as an internal control for normalization. Relative expression was quantified as fold changes using the 2^−∆∆CT^ method. The control group served as the calibrator for relative quantification against the 100, 200, and 400 ppm treatment groups at 12, 24, and 72 h post-exposure. Statistical analyses of qPCR data were performed using ∆CT values. For qPCR analysis, each biological replicate was analyzed in technical triplicate.

### 2.3. Histopathological Analysis

For histopathological analysis, three olive flounders in the control and experimental groups (100, 200, and 400 ppm) were collected at 12, 24, and 72 h post-exposure. Harvested gill and liver samples were fixed in 10% neutral buffered formalin (Sigma-Aldrich). The fixed tissues were processed using a tissue processor (Leica TP1020, Leica Biosystems, Wetzlar, Germany) for dehydration, clearing, and paraffin infiltration, and then embedded in paraffin wax using a paraffin embedding station (Leica EG1150, Leica Biosystems). Tissue blocks were sectioned at 5 µm using a microtome (HistoCore AUTOCUT, Leica Biosystems) and stained with hematoxylin and eosin (H&E). Three representative tissue sections from each fish and five microscopic fields per section were examined using a microscope (Axioscope 5 MAT, Carl Zeiss, Oberkochen, Germany).

### 2.4. Statistical Analysis

All data were analyzed using SPSS (version 25.0; IBM Corp., Armonk, NY, USA). Significant differences were identified by two-way ANOVA, followed by Tukey’s multiple comparison test as a post hoc test. Statistical significance was set at *p* < 0.05.

## 3. Results

### 3.1. Gene Expression Analysis

#### 3.1.1. Drug Metabolism-Related Gene Expression

**Figure 1:**
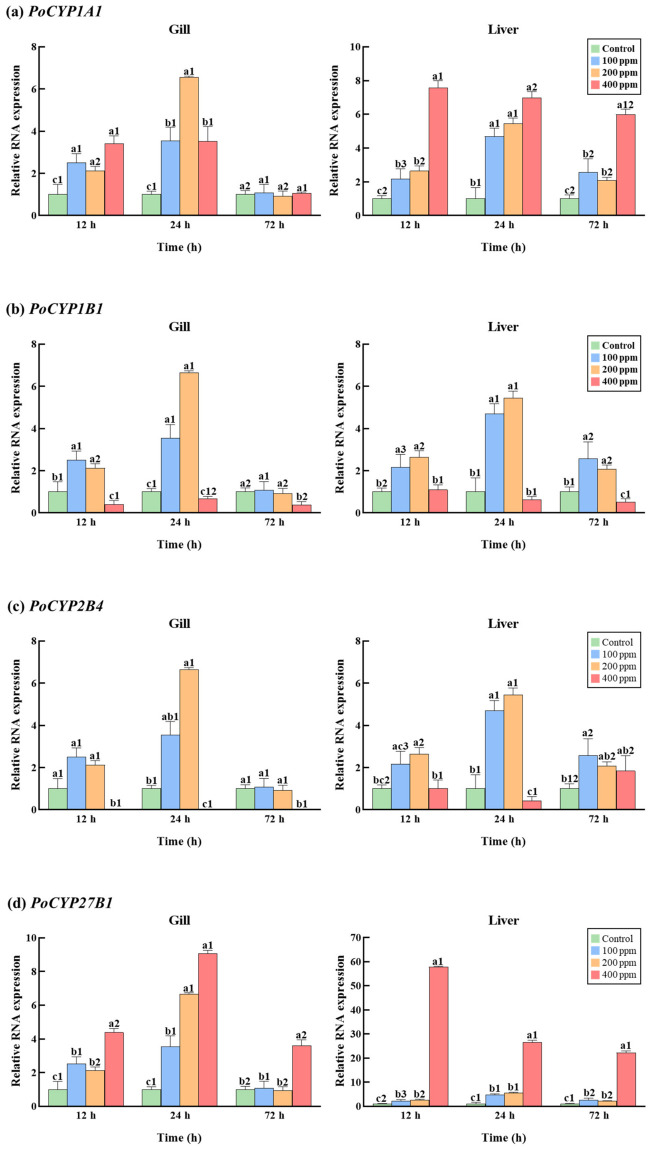

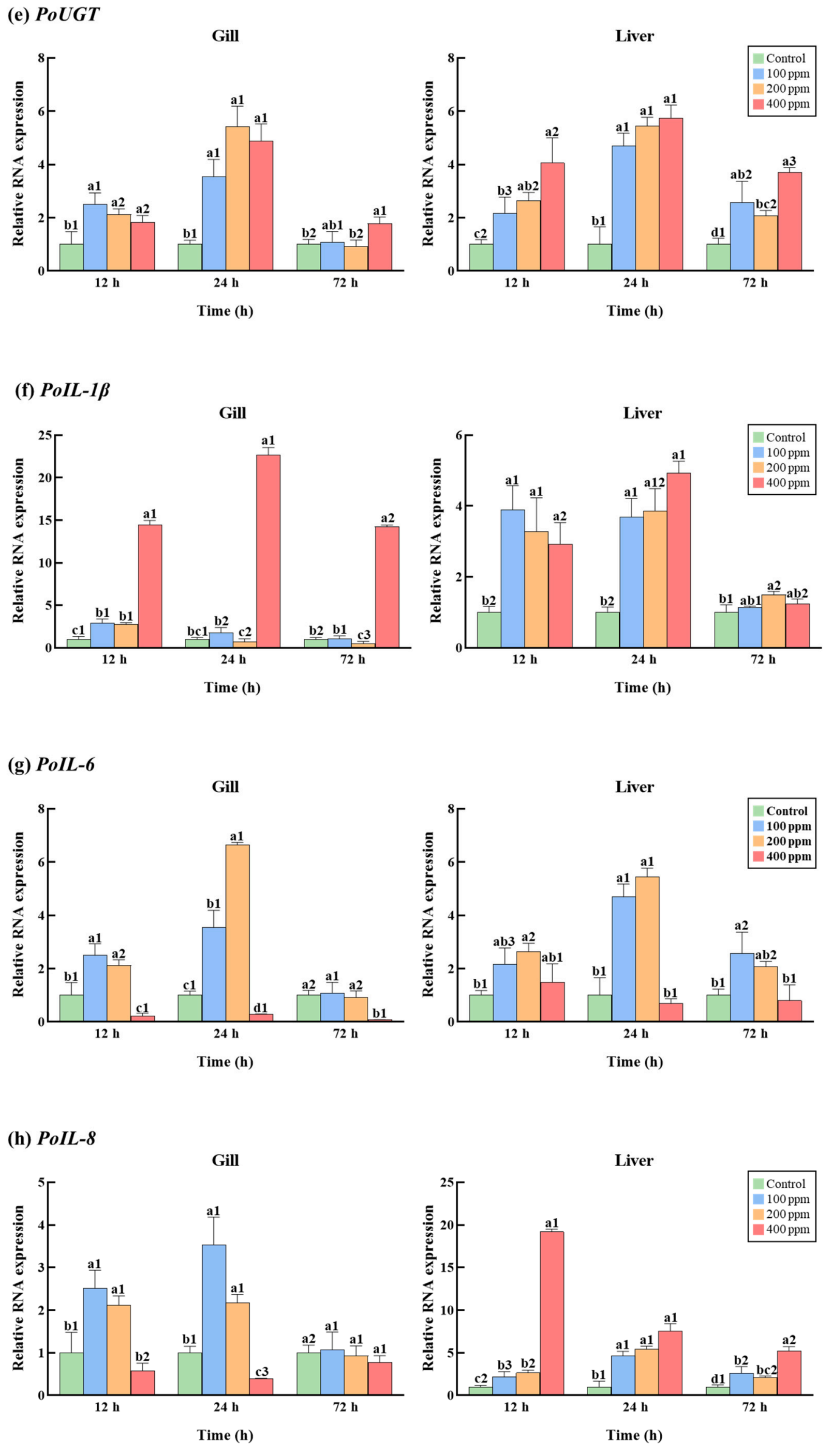

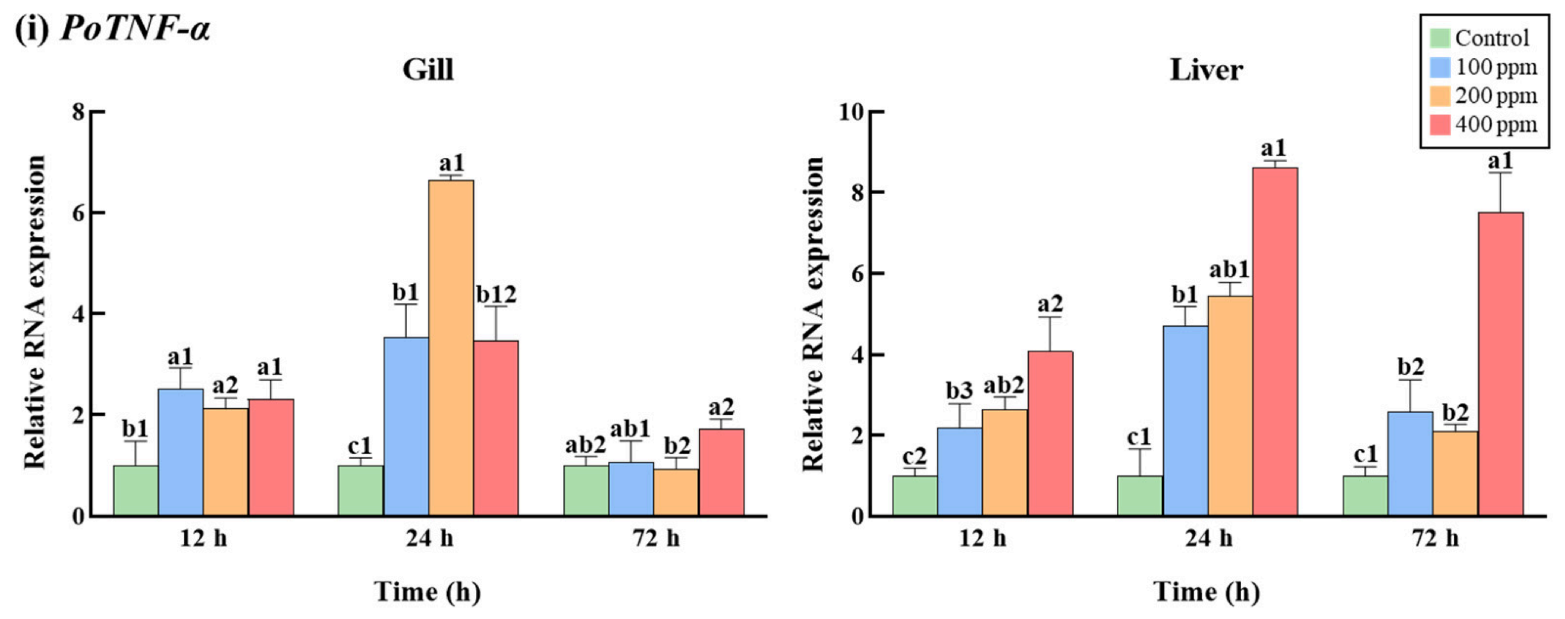
Gene expression of *PoCYP1A1* (**a**), *PoCYP1B1* (**b**), *PoCYP2B4* (**c**), *PoCYP27B1* (**d**), *PoUGT* (**e**), *PoIL-1β* (**f**), *PoIL-6* (**g**), *PoIL-8* (**h**), *PoTNF-α* (**i**) in the gills and liver of olive flounder at 12, 24, and 72 h after exposure to different concentrations of OTC. Error bars indicate the standard deviation of three biological replicates. Different letters indicate significant differences among OTC concentrations at the same sampling time, whereas different numbers indicate significant differences among sampling times within the same OTC concentration (*p* < 0.05).

In the gills, the expression of drug metabolism-related genes in the 100 and 200 ppm groups initially increased at 12–24 h after OTC exposure and then decreased at 72 h (Figure 1a–e). *PoCYP1A1* expression significantly increased (*p* < 0.05) by 2.5-, 2.1-, and 3.4-fold at 12 h in the 100, 200, and 400 ppm groups, respectively, compared with the control. The expression peaked at 24 h in the 200 ppm group (6.6-fold) and then returned to levels comparable to those in the control at 72 h (Figure 1a).

Similarly, *PoCYP1B1* and *PoCYP2B4* expression peaked at 24 h in the 200 ppm group (6.6-fold) compared with the control (Figure 1b,c). By contrast, *PoCYP27B1* expression treatment dependently increased by 3.5-, 6.6-, and 9.1-fold at 24 h in the 100, 200, and 400 ppm groups, respectively. At 72 h, the expression levels in the 100 and 200 ppm groups decreased to levels comparable to those in the control, whereas those in the 400 ppm group still showed 3.6-fold higher expression (Figure 1d). *PoUGT* expression also peaked at 24 h, with 3.5-, 5.5-, and 4.9-fold increases in the 100, 200, and 400 ppm groups, respectively. At 72 h, only the 400 ppm group maintained significantly higher expression than the control (Figure 1e).

In the liver, the expression of drug metabolism-related genes was generally upregulated after OTC exposure (Figure 1a–e). *PoCYP1A1* expression significantly increased (*p* < 0.05) by 2.2-, 2.6-, and 7.6-fold at 12 h in the 100, 200, and 400 ppm groups, respectively, compared with the control. This elevated expression persisted up to 24 h, and the 100, 200 and 400 ppm groups maintained higher levels than the control even at 72 h (Figure 1a). A similar trend was observed for *PoCYP1B1*, with significant increases (*p* < 0.05) in the 100 and 200 ppm groups from 2.2- and 2.6-fold at 12 h to 4.7- and 5.4-fold at 24 h, respectively (*p* < 0.05; Figure 1b). *PoCYP2B4* expression reached its maximum in the 200 ppm group at 24 h (5.4-fold) (Figure 1c). By contrast, *PoCYP27B1* expression exhibited a markedly stronger response in the 400 ppm group, with expression increasing dramatically at 12 h (58-fold) and remaining elevated at 24 and 72 h (27- and 22-fold, respectively), representing the highest level among the analyzed genes (Figure 1d). Meanwhile, *PoUGT* expression treatment dependently increased by 4.7-, 5.4-, and 5.7-fold in the 100, 200, and 400 ppm groups, respectively, and peaked at 24 h in all exposure groups compared with the control (Figure 1e).

#### 3.1.2. Immune-Related Gene Expression

In the gills, the expression of inflammatory cytokine genes initially increased at 12–24 h and then decreased at 72 h (Figure 1f–i). Among the analyzed genes, *PoIL-1β* exhibited the highest induction, particularly in the 400 ppm group, where its expression increased by 14.5-, 22.7-, and 14.2-fold at 12, 24, and 72 h, respectively. By contrast, their expression levels in the 100 and 200 ppm groups remained comparable to those in the control (Figure 1f). *PoIL-6* expression significantly increased in the 100 and 200 ppm groups at 12 and 24 h (*p* < 0.05), reaching 3.5- and 6.6-fold at 24 h, respectively (Figure 1g). A comparable expression pattern was observed for *PoIL-8*, although no significant differences were detected among the treatment groups at 72 h (Figure 1h). *PoTNF-α* expression also showed a similar temporal pattern to *PoIL-8*, with elevated levels at 12–24 h and a peak in the 200 ppm group at 24 h (6.6-fold), followed by a reduction across all groups at 72 h (Figure 1i).

In the liver, the expression of immune-related genes also increased after OTC exposure (Figure 1f–i). *PoIL-1β* expression peaked at 24 h in the 400 ppm group (5.0-fold) and then decreased at 72 h (Figure 1f). *PoIL-6* expression also peaked at 24 h, with 4.7- and 5.4-fold increases in the 100 and 200 ppm groups, respectively (Figure 1g). By contrast, *PoIL-8* exhibited the highest expression at 12 h in the 400 ppm group (19.2-fold), followed by a gradual decrease over time (Figure 1h). A comparable pattern was observed for *PoTNF-α*, with expression increasing after OTC exposure and reaching its maximum at 24 h in the 400 ppm group (8.6-fold) (Figure 1i).

### 3.2. Histopathological Analysis

**Figure 2:**
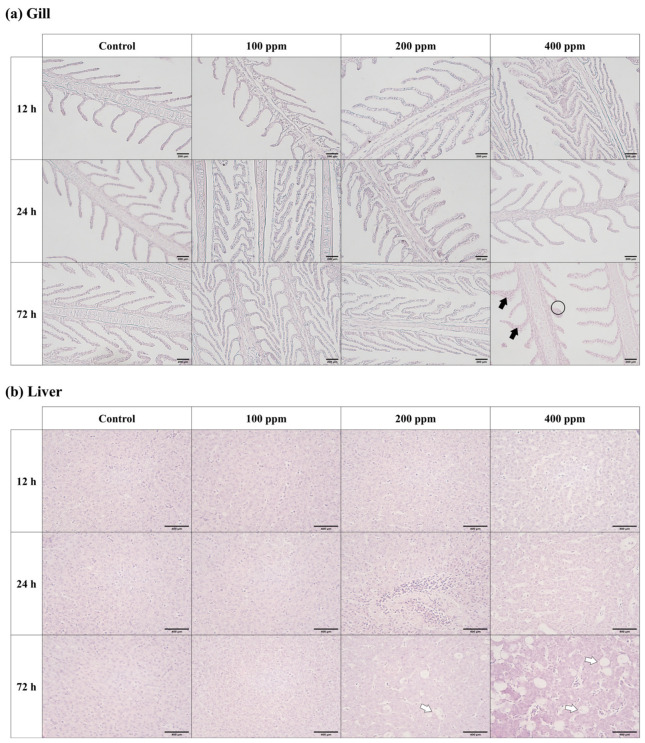
Histopathological changes in gills (**a**) and liver (**b**) at 12, 24, and 72 h after OTC exposure to olive flounder. Black arrows: epithelial cell edema; black circles: aneurysm; white arrows: phagocytosis.

Histopathological examination of the gills revealed no histopathological changes in the 100 and 200 ppm groups compared with the control group at all time points. At 12 and 24 h, no histopathological changes in the gills were observed in the 400 ppm group compared with the control group. However, epithelial cell edema (black arrows) and mild aneurysm (black circles) were observed at 72 h (Figure 2a). At 12 and 24 h, no histopathological changes in the liver were observed in the 100, 200, and 400 ppm groups compared with the control group. However, increased macrophages with activated phagocytosis (white arrows) were observed at 72 h in the 200 and 400 ppm groups (Figure 2b).

## 4. Discussion

In this study, the expression of drug metabolism- and immune-related genes was analyzed in the gills and liver of olive flounder exposed to OTC at various concentrations. In addition, histopathological analysis was performed to evaluate the effects of OTC exposure.

OTC can be administered to fish via oral, injection, or immersion methods. However, oral administration may be unreliable in diseased or stressed fish because of reduced feed intake, resulting in inconsistent dosing [28]. Although injection ensures accurate delivery of a correct dosage calculation, it is labor-intensive and may induce handling stress [29]. By contrast, immersion allows a large number of fish to be exposed simultaneously and is independent of feeding behavior, making it a practical and less stressful approach in aquaculture and experimental settings [30]. Therefore, immersion was employed in this study.

Chemical exposure in fish alters the expression of *CYP* enzymes and cytokines, which play critical roles in drug detoxification and tissue damage regulation [31,32]. The expression of *CYP1* and *CYP2* family genes is upregulated in the livers of rainbow trout and zebrafish exposed to compounds such as phenobarbital [33,34]. Similarly, OTC exposure in the present study induced the upregulation of *PoCYP1A1* and *PoCYP27B1* expression in all treatment groups, whereas *PoCYP1B1* and *PoCYP2B4* expression levels were upregulated only in the 100 and 200 ppm groups (Figure 1a–e).

The upregulation of *PoCYP1A1* and *PoCYP1B1* expression can be explained by the characteristic mechanism of the *CYP1* family, in which xenobiotic compounds diffuse into cells, bind to intracellular carrier proteins, and subsequently translocate to the nucleus to activate gene transcription (Figure 1a,b) [35]. Meanwhile, *CYP2* family genes are regulated by the xenobiotic receptor Pregnane X receptor (PXR) in zebrafish and humans [36,37,38], indicating that the observed changes in *PoCYP2B4* may be associated with PXR-mediated regulation (Figure 1c).

The *CYP27* family is involved in the biosynthesis of steroid hormones, including estrogen, in mice [39]. These hormones influence the metabolism and detoxification of xenobiotics in the liver [40]. In the present study, *PoCYP27B1* expression was markedly increased in the liver compared with the gills after OTC exposure (Figure 1d). Although CYP27 family genes are known to be associated with steroid hormone biosynthesis, the relationship between *PoCYP27B1* expression, steroid hormone biosynthesis, and drug metabolism remains unclear and requires further investigation.

*CYP* genes are predominantly expressed in the liver, although higher expression levels in the gills have also been reported [35]. In the present study, the same genes exhibited different expression patterns between the gills and liver, which may be attributed to tissue-specific differences in the distribution of ATP-binding cassette (ABC) transporters. ABC transporters can transport and limit the distribution of xenobiotics, including drugs, and their distribution varies among different internal organs. Therefore, these differences may have contributed to the observed tissue-specific variation in gene expression between the liver and gills [41,42].

*UGT* plays a key role in phase II metabolism by facilitating the excretion of metabolites generated during *CYP*-mediated phase I reactions. *PoUGT* expression significantly increased after OTC exposure (Figure 1e), suggesting that phase I (*CYP*) and phase II (*UGT*) pathways are functionally linked. However, previous studies have reported decreased *UGT* expression in olive flounder exposed to amprolium hydrochloride [43]. This discrepancy indicates that *UGT* expression may vary depending on the type of compound and its mechanism of action [43,44].

Transcriptome-based studies have demonstrated that xenobiotic exposure affects not only drug metabolism-related genes such as *CYP* and *UGT* but also immune-related genes in olive flounder [25]. Cytokines, including *PoIL-1β*, *PoIL-6*, *PoIL-8*, and *PoTNF-α*, were upregulated after OTC exposure (Figure 1f–i), suggesting that detoxification processes and immune responses may be simultaneously modulated. Among these cytokines, *IL-1β* is a representative pro-inflammatory mediator in vertebrates and plays a key role in early immune responses in fish [26,45,46]. A rapid increase in *PoIL-1β* levels was observed within 24 h in both the gills and liver, indicating its involvement in early immune activation (Figure 1f). *IL-6* exerts both pro- and anti-inflammatory functions [47]. In the present study, *IL-6* levels increased in the 100 and 200 ppm groups, whereas the 400 ppm group showed levels comparable to those in the control (Figure 1g), suggesting that immune regulation may differ under high-concentration exposure. *IL-8*, an immunoregulatory cytokine responsive to immune cell activation and cytokine signaling [48,49], increased by 19.2-fold in the liver of the 400 ppm group compared with the control (Figure 1h). *TNF-α*, which is involved in immune regulation and inflammatory signaling [50,51], also showed increased levels after OTC exposure. This response may be associated with coordinated interactions with *IL-1β*, *IL-6*, and *IL-8*, suggesting that cytokine responses function as an interconnected regulatory network rather than independent pathways (Figure 1i).

Notably, the expression patterns of *CYP*, *UGT*, and cytokine genes did not exhibit a clear treatment-dependent trend. Similar studies have reported that gene expression responses to OTC exposure may vary depending on treatment conditions and sampling time, rather than exhibiting a consistent treatment-dependent trend [52]. In addition, drug responses can vary depending on genetic and environmental factors, as well as exposure duration [53]. Although short-term exposure is typically associated with increased *CYP* expression, chronic exposure may lead to decreased expression as part of an adaptive response to contaminated environments [54].

Exposure to pharmaceuticals and other chemicals can induce organ-level adverse effects in fish [55], and histopathological analysis is widely used to assess such impacts in aquatic organisms [56]. In rainbow trout (*Oncorhynchus mykiss*), acute OTC exposure induces edematous and aneurysmal changes in the gills and inflammatory responses, including leukocyte infiltration, in the liver [57]. In olive flounder, similar histopathological alterations were observed in the 400 ppm group, including cellular edema and mild aneurysms in the gills and increased macrophages exhibiting enhanced phagocytic activity in the liver, suggesting that high-concentration OTC exposure may induce tissue-level alterations. Together with the marked changes in the expression of drug metabolism- and immune-related genes, these histopathological findings suggest that exposure to excessive concentrations of OTC may impose physiological stress on olive flounder. Therefore, the responses observed in the 400 ppm group should not be interpreted solely as enhanced metabolic or immune activation, but may also indicate tissue stress associated with high-dose OTC exposure.

Overall, these findings demonstrate that OTC exposure alters the expression of genes associated with drug metabolism and immune responses in olive flounder, highlighting their involvement in early immune activation and detoxification. Although the present study provides valuable information regarding the molecular responses of olive flounder to OTC exposure, further studies incorporating protein-level analyses are needed to better understand the relationship between gene expression and biological function and to clarify the physiological significance of the observed molecular changes. These results provide fundamental insights into the molecular and physiological responses of olive flounder to OTC exposure.

## 5. Conclusions

OTC exposure modulates drug metabolism- and immune-related responses in olive flounder, as evidenced by altered expression of *CYP*, *UGT*, and cytokine genes. In addition, histopathological alterations observed at higher exposure concentrations suggest that excessive OTC exposure may induce tissue-level damage. Therefore, to ensure the health and safety of aquaculture organisms, the use of high concentrations of OTC should be discouraged, and adherence to appropriate usage levels and management practices is essential. These findings provide insights into the effects of OTC exposure on drug metabolism and toxicity in olive flounder.

## Figures and Tables

**Table 1:** Primers used for RT-qPCR in this study.

Genes	Primer	Sequences (5′–3′)	Reference
*β-actin*	forward	TGATGAAGCCCAGAGCAAGA	[22]
	reverse	CTCCATGTCATCCCAGTTGGT	
*PoCYP1A1*	forward	GATGAGGAGCTGTGGAAAGA	[23]
	reverse	AGACTTCATTTCGAGCGATG	
*PoCYP1B1*	forward	GTGACTCTGCTCTTCTCCCTC	[24]
	reverse	GTACTGGAAAGAGGTGAAGTCG	
*PoCYP2B4*	forward	CACACATACAAGAGCGTTGC	[23]
	reverse	CCCATGAGCTCTGTGTCTTT	
*PoCYP27B1*	forward	CCTCGTAGAGTTCGATGTGA	[25]
	reverse	CATCGCTCGATAAACTGGAG	
*PoUGT*	forward	AGAAGGGCAACTGCAAAGAC	[25]
	reverse	GTTGAGTCGGCTTCAGTCAA	
*Po* *IL-1β*	forward	GACAGTGAGATGGTGCGATTTC	[25]
	reverse	ACCATCACTGGCCTGTTGTCT	
*Po* *IL-6*	forward	CTCCAAACACAATGCCGACTT	[26]
	reverse	CTCCTGCTCCTCACCTGAAAA	
*Po* *IL-8*	forward	GGCTCCGTGGGTGAAGAGAGTCATC	[27]
	reverse	TCAAACAAACACATTAGGGTCGT	
*Po* *TNF-α*	forward	TCCCACGAAGCGGCCTCTACTT	[27]
	reverse	TGCCCAGGGACTCCGTGAAGAGC	

## Data Availability

The data presented in this study are available upon request from the corresponding author.
